# Supplementary figures and images for: Sox10-Deficient Drug-Resistant Melanoma Cells Are Refractory to Oncolytic RNA Viruses
Source: Cells. 2023 Dec 29;13(1):73. doi: 10.3390/cells13010073 (PMC10777920; doi:10.3390/cells13010073)

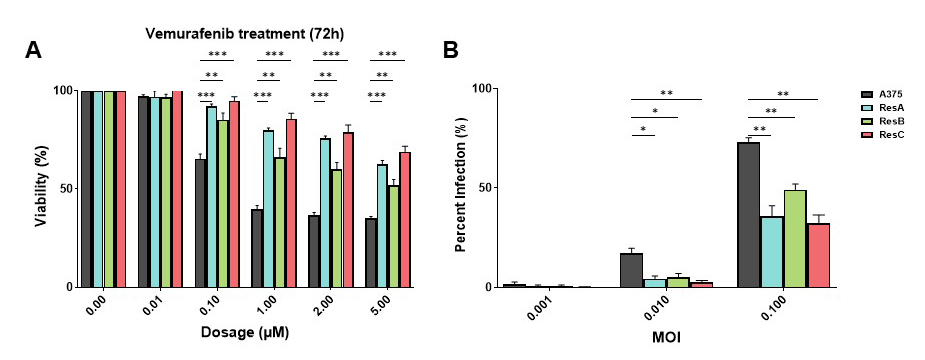

Supplement: Supplementary file 1 [file cells-13-00073-s001.zip › Suppl FigS1.tif]

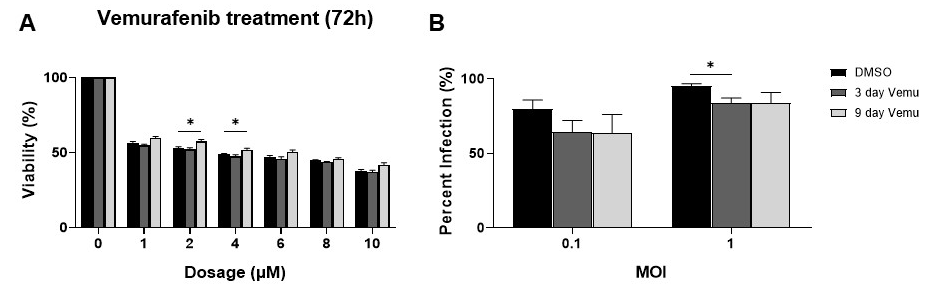

Supplement: Supplementary file 1 [file cells-13-00073-s001.zip › Suppl FigS2.tif]

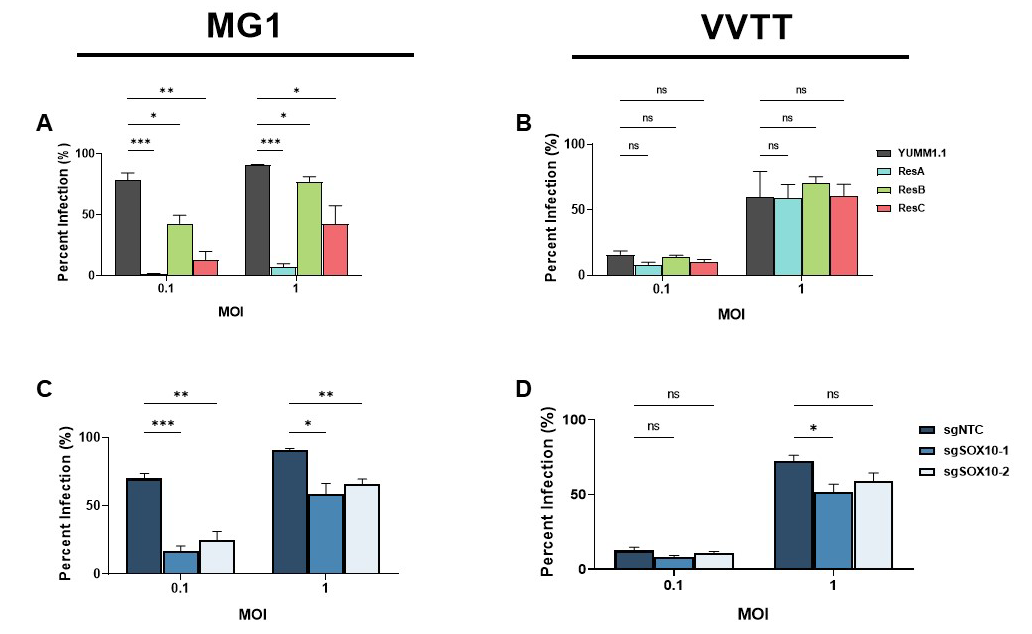

Supplement: Supplementary file 1 [file cells-13-00073-s001.zip › Suppl FigS3.tif]

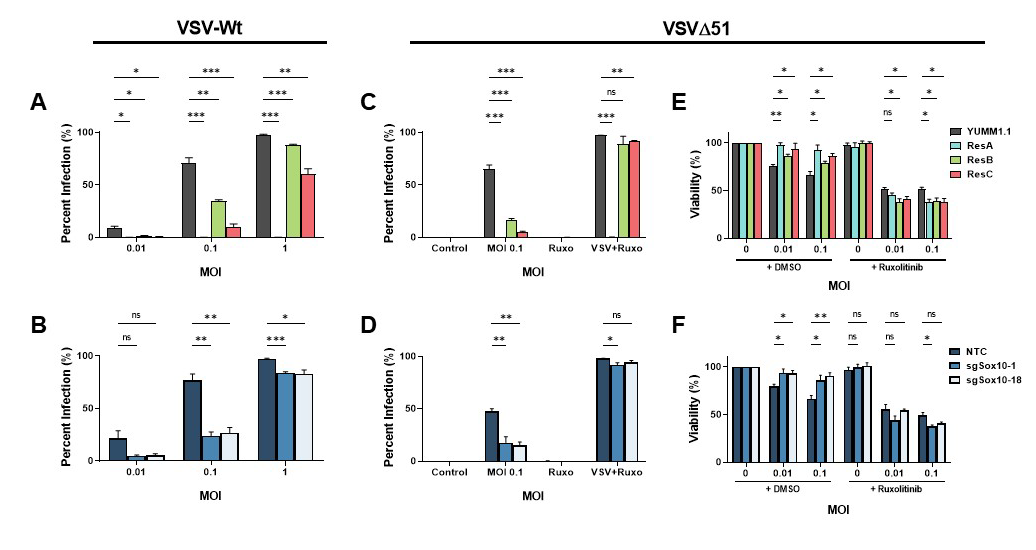

Supplement: Supplementary file 1 [file cells-13-00073-s001.zip › Suppl FigS4.tif]

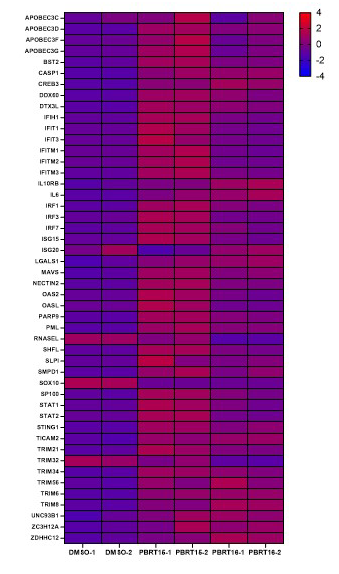

Supplement: Supplementary file 1 [file cells-13-00073-s001.zip › Suppl FigS5.tif]
